# Trimebutine Promotes Glioma Cell Apoptosis as a Potential Anti-tumor Agent

**DOI:** 10.3389/fphar.2018.00664

**Published:** 2018-06-21

**Authors:** Yi-pu Fan, Pei Liu, Wei-kang Xue, Wei-jiang Zhao, Hong-chao Pan

**Affiliations:** ^1^Center for Neuroscience, Shantou University Medical College, Shantou, China; ^2^Guangdong Provincial Key Laboratory for Breast Cancer Diagnosis and Treatment, Cancer Hospital of Shantou University Medical College, Shantou, China

**Keywords:** trimebutine, glioma/glioblastoma, apoptosis, p-AKT, p-ERK

## Abstract

Gliomas are the most common primary brain tumors with a usually fatal malignancy. They are associated with a poor prognosis although multiple therapeutic options have been available. Trimebutine is one of the prokinetic agents and it has been mainly used for treatment of disorders of the gastrointestinal (GI) tract such as irritable bowel syndrome. However, its effects on glioma cells remain unknown. Here, we used various concentrations of trimebutine to treat SHG44, U251, and U-87 MG human glioma/glioblastoma cells. And combined experiments of MTT, colony formation assay, and wound healing assay, as well as western blot and immunofluorescence staining were used to evaluate the effects of trimebutine on glioma cells. The results demonstrated that trimebutine significantly inhibited cell viability and colony formation. A significant inhibition of glioma cell migration was also indicated by wound healing assay. In addition, trimebutine promoted cell apoptosis and induced Bcl-2 downregulation, accompanied with Bax upregulation. Both immunofluorescence staining and western blot results showed that trimebutine increased the level of active Caspase-3. Moreover, trimebutine reduced the activation of both AKT and ERK signaling pathways. In subcutaneous U-87 MG cell xenograft tumors in nude mice, trimebutine significantly inhibited tumor growth. More TUNEL-positive apoptotic cells in tumor sections were observed in trimebutine-treated mice when compared to the vehicle control. Reduced Bcl-2 and upregulated Bax, as well as perturbed p-AKT and p-ERK signaling pathways were also observed in trimebutine-treated xenograft tissues. Our combined data indicated that trimebutine may be potentially applied for the clinical management of glioma/glioblastoma.

## Introduction

Glioblastoma multiforme (GBM) is one of the most malignant and lethal brain tumors, accounting for 70% of human primary malignant brain tumors (Maher et al., 2001; Wen and Kesari, 2008). Glioblastoma is a grade IV astrocytoma and the current treatment for glioblastoma patients is mostly dependent on surgery resection in combination with radiotherapy and chemotherapy (Louis et al., 2007). However, radiation and chemotherapy only lead to a 9–12 months of median survival time among patients with GBM (Louis et al., 2007; Chen et al., 2012). Therefore, it is urgent to understand the mechanisms underlying GBM tumorigenesis and to seek effective therapeutic management for glioma.

Trimebutine maleate is an opioid agonist that acts on the peripheral delta, mu and kappa receptors (Kaneto et al., 1990; Jhee et al., 2007). It has been widely applied in the treatment of irritable bowel syndrome and other gastrointestinal (GI) disorders (Delvaux and Wingate, 1997; Poynard et al., 2001). Trimebutine maleate is active in counteracting local inflammation and stress-induced rectal hyperalgesia in rats (Lacheze et al., 1998). In addition, trimebutine has been reported to regulate GI tracts through a Ca^2+^ antagonist-like action by inhibiting the influx of extracellular Ca^2+^ into the smooth muscles (Shimada et al., 1990; Nagasaki et al., 1991). Furthermore, one report demonstrated that trimebutine can dose-dependently induce apoptosis in human LOVO colon carcinoma cells and inhibited cell proliferation by inactivating the ERK1/2 signaling pathway (Jemel-Oualha et al., 2016). In contrast, the therapeutic effects of trimebutine on gliomas have not been reported. Here, we showed that trimebutine can inhibit glioma cell viability and migration, and induce apoptosis in glioma cells. We also showed that trimebutine can inactivate the PI3K/AKT and Ras/MAPK signaling pathways in three human glioma/glioblastoma cell lines. Furthermore, trimebutine significantly suppressed human U-87 MG glioblastoma xenograft growth *in vivo* in a nude mouse model.

## Materials and Methods

### Cells and Reagents

Normal human astroglia HEB cell line, SHG44 and U251 human glioma, and U-87 MG human glioblastoma cell lines were purchased from the Chinese Type Culture Collection (CTCC, Shanghai, China) and were maintained in Dulbecco’s modified Eagle’s medium low Glucose (DMEM, Thermo Scientific HyClone, Beijing, China) supplemented with 50 U/mL of a penicillin/streptomycin mixture (Solarbio Biotech Corp., Beijing, China) and 10% fetal bovine serum (Sijiqing Biotech Corp., Hangzhou, China). The cells were routinely grown in 60-cm^2^ cell culture plates (Corning Inc., Corning, NY, United States) at 37°C in a humidified atmosphere with 5% carbon dioxide. Trimebutine (#K1313, sc-204928) was obtained from Santa Cruz Biotechnology, Dallas, TX, United States. MTT and TUNEL assay kits were purchased from Beyotime Biotechnology, Jiangsu, China.

### MTT Assay

HEB and SHG44, U251, and U-87 MG cells were seeded onto a 96-well plate at a density of 3 × 10^3^ cells per well. After overnight incubation, the culture medium was aspirated. For the determination of the IC50 values, HEB cells were treated with trimebutine dosed from 0 to 1000 μM in complete culture medium, while SHG44, U251, and U-87 MG cells were incubated with trimebutine at doses ranging from 0 to 400 μM in complete culture medium for 48 h. To further evaluate the effect of trimebutine on glioma/glioblastoma cell viability, SHG44, U251, and U-87 MG cells were incubated with trimebutine at doses ranging from 0 to 200 μM in complete culture medium for 24, 48, and 72 h, respectively. Cells in the vehicle control group were treated with dimethyl sulphoxide (DMSO; 0.1%). At each destined time point, 10 μl of MTT (5 mg/ml; Beyotime, Jiangsu, China) was added to each well. Cells were further cultured for 4 h. Then, the culture medium was removed, and 100 μl of DMSO was added. The absorbance was measured at a wavelength of 490 nm by an ELISA plate reader (Infinite M1000, Tecan, Switzerland). The cell survival rate was determined with the formula: Survival rate (%) = mean OD_treated groups_/OD_vehicle control group_. The half-maximal inhibitory concentration (IC50) at 48 h was calculated with the survival of vehicle-treated cells set at 100%.

### Wound Healing Assay

U-87 MG cells were seeded at a density of 5 × 10^4^ cells per well in 96-well plates in complete cell culture medium. After treatment with various concentrations of trimebutine, the monolayer of cells was scratched with a 10 μl plastic pipette tip to create a uniform wound. The wound width was then examined after 0, 24, 48, and 72 h of incubation under a phase-contrast microscope at ×100 magnification (Olympus, IX51, Japan). Photographs of at least three random fields were taken, and the cell migration ability was expressed by the closure of the gap distance.

### Colony Formation Assay

SHG44, U251 and U-87 MG cells (1500 cells/well) were seeded onto a 24-well plate. After treatment with Trimebutine at 37°C for 10 days, the colonies were fixed with methanol for 20 min, stained with 0.1% crystal violet, and visualized under a phase-contrast light microscope (Olympus, IX51, Japan). An accumulated growth of more than 50 cells was identified as the formation of a colony.

### Flow Cytometry Assay of Cell Apoptosis

SHG44, U251 and U-87 MG cells were seeded at a density of 5 × 10^5^ cells per well onto 6-well plates in complete culture medium. After overnight incubation, the culture medium was removed, and the cells were incubated with trimebutine at doses ranging from 0 to 200 μM in complete culture medium. And cells were further cultured for 48 h. To further evaluate apoptosis after treatment with trimebutine, the Annexin V-EGFP/PI (Propidium Iodide) Apoptosis Kit was employed according to the manufacturer’s protocol (Beyotime, Jiangsu, China). Cell apoptosis was measured using Accuri C6 Cytometer (BD Biosciences, San Jose, CA, United States). Annexin V-EGFP/PI was excited with 495 and 535 nm lasers and the signals were detected using 530/30 and 585/40 band pass (BP) filters, respectively.

### Cell Immunofluorescence Staining

U-87 MG cells were seeded on a cover slide in a 24-well plate at a density of 5 × 10^4^ cells per well. After overnight incubation, the culture medium was removed, and the cells were incubated with trimebutine at doses ranging from 0 to 200 μM in complete culture medium. And cells were further cultured for 48 h. Fixed U-87 MG cells were incubated with rabbit anti-human active Caspase-3 antibody (1:200; #BM3937, Boster, Wuhan, China) overnight at 4°C. After washing in PBS three times for 5 min each, the samples were incubated at room temperature with donkey anti-rabbit secondary antibody conjugated to Dylight^TM^ 594 (1:500; Jackson ImmunoResearch Inc, West Grove, PA, United States) for 60 min. The cells were then stained with 4′, 6-diamidino-2-phenylindole (DAPI) and mounted using anti-fade mounting solution (Beyotime).

### Western Blot Analysis

Equivalent quantities of either cell lysates from cells or tissue lysates from xenograft tumor tissues were heated at 100°C in 20% sample loading buffer (0.125 mol/L Tris-HCl, pH 6.8, 20% glycerol, 10% sodium dodecyl sulfate, 0.1% bromophenol blue, and 5% β-mercaptoethanol), resolved by 10% sodium dodecyl sulfate polyacrylamide gel electrophoresis and electroblotted onto polyvinylidene difluoride membranes (Millipore, Billerica, MA, United States). Non-specific protein binding sites were blocked with 5% BSA diluted in Tris-buffered saline buffer containing 0.05% Tween-20 (TBST, pH 7.4). The membranes were incubated with antibodies specific for ERK (MK1; 1:1000; #sc-135900), AKT G-5; 1:1000; #sc-53373), p-ERK (E-4; 1:1000; #sc-7383), p-AKT (11E6; 1:1000; #sc-81433), Bcl-2 (C-2; 1:1000; #sc-7382), Bax (C-19; 1:1000; #sc-526), GAPDH (2E3-2E10; 1:1000; #sc-293335), β-actin (C-4; 1:1000; #sc-47778) (all these antibodies were obtained from Santa Cruz Biotechnology, Inc., Dallas, TX, United States), and an antibody targeting both pro- and cleaved Caspase-3 (1:500; #BM3257, Boster), overnight at 4°C. After three washes for 5 min each, horseradish peroxidase (HRP)-conjugated goat anti-mouse (#BA1051) and goat anti-rabbit (#BA1055) secondary antibodies (1:1000 for both; Boster) diluted in 5% BSA in TBST were applied, followed by three washes with TBST for 5 min each at room temperature. The signal intensities were quantified using Image J software (NIH) as the average densitometry multiplied by the area (measured as the number of pixels).

### Xenograft Nude Mice Model

Four-week old female nude mice were purchased from the Model Animal Research Center of Nanjing University and maintained at the Animal Facility of Shantou University Medical College for 1 week prior to experimental use. U-87 MG cells (5 × 10^5^ cells/mouse) were subcutaneously injected into the mice. After the tumors reached a volume of 2 mm^3^, the nude mice were randomized into two groups (*n* = 5 per group). Then, Trimebutine (100 mg/kg/day) or DMSO was injected intraperitoneally into the mice everyday for 7 days. The tumor size was measured using a vernier caliper at days 7, 10, 14, 16, 18, and 21. Tumor volume was estimated by the formula (L × S^2^/2, where L: longest diameter and S: shortest diameter). The mice were sacrificed and the tumors were harvested and weighed using an electronic balance (ME104, Mettler Toledo, Shanghai, China). The experiments were approved by the Institutional Animal Care and Use Committee of Shantou University Medical College.

### H&E Staining and Immunofluorescence Tissue Staining

The tumor tissues were cryo sectioned in 8 μm for H&E staining as a routine based on previous work (Zhao et al., 2009). The cell apoptosis in xenograft tumor tissues was co-evaluated by both PI staining and terminal deoxynucleotidyl transferase (TdT) deoxyuridine 5-triphosphate dUTP Nick- End Labeling (TUNEL) staining (Beyotime). The tissue slides were washed using PBS and incubated with TF3-dUTP and PI for 60 min at 37°C. Both TUNEL-positive and PI-positive cells were visualized using a fluorescence microscope (Zeiss, Axio Imager A2, Germany). Photographs of at least five random fluorescent fields were taken.

### Statistical Analysis

Statistical analyses were performed using SPSS (Statistical Package for the Social Sciences) 19.0 software (SPSS, Chicago, IL, United States). The intensities were quantified using Image J software (NIH). The data are expressed as the means ± SEM of at least 3–4 independent experiments and were compared using either two-tailed independent Student’s *t*-test or one-way ANOVA with Tukey’s *post hoc* test for multiple comparisons. Differences were considered to be significant at *P* < 0.05.

## Results

### Trimebutine Inhibits the Viability of Glioma Cells

**FIGURE 1:**
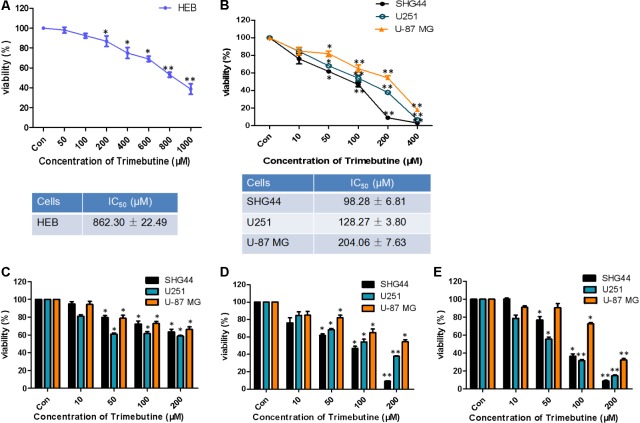
Effect of trimebutine on glioma/glioblastoma cell viability. IC50 values in either normal human astroglia HEB cell line **(A)** or the three glioma/glioblastoma cell lines **(B)**. SHG44 and U251 human glioma and U-87 MG human glioblastoma cells were treated with various concentrations of trimebutine for 24 **(C)**, 48 **(D)**, and 72 h **(E)**. MTT assay was used to assess cell viability. The data are presented as the means ± SEM from 3 independent experiments. ^∗^*P* < 0.05 and ^∗∗^*P* < 0.01 vs. vehicle control (one-way ANOVA with Tukey’s *post hoc* test).

The IC50 value for trimebutine at 48 h was 862.30 ± 22.49 μM in normal human astroglia HEB cells (**Figure 1A**), whereas the IC50 values for trimebutine at 48 h in SHG44, U251, and U-87 MG cells were 98.28 ± 6.81 μM, 128.27 ± 3.80 μM, and 204.06 ± 7.63 μM, respectively (**Figure 1B**). We found that trimebutine can significantly reduce the cell viability compared with the vehicle control in both dose-dependent and time-dependent manners (**Figures 1C–E**). The results demonstrated that trimebutine can significantly inhibit glioma/glioblastoma cell viability while exerting no apparent toxic effect on normal human astroglia cells.

### Trimebutine Inhibits Cell Migration

**FIGURE 2:**
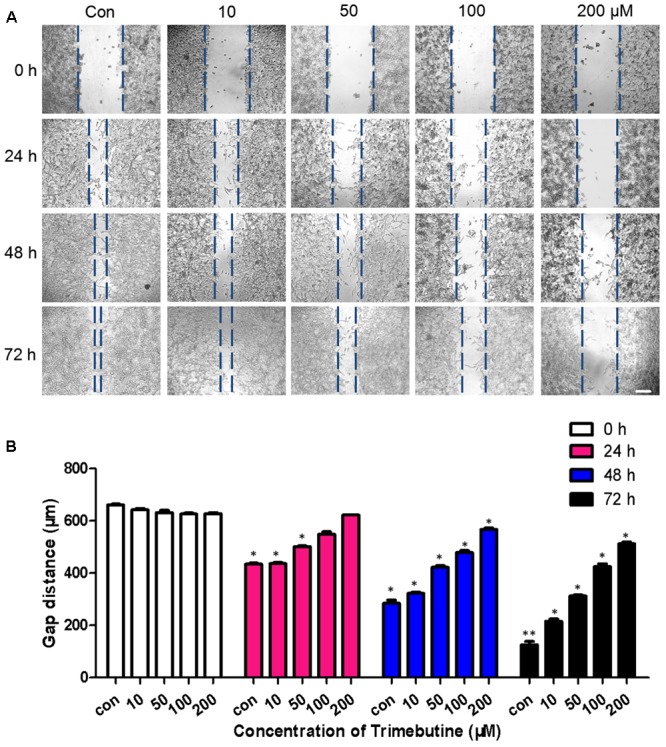
Trimebutine suppresses U-87 MG cell migration in a wound healing assay. **(A)** Representative images captured under a phase contrast microscope after 24, 48, and 72 h of treatment with various concentrations of trimebutine. The vertical lines indicate the wound edge. Scale bar, 200 μm. **(B)** The gap distance was measured with Image J, and the average gap distances are shown. The data are presented as the means ± SEM for 4 independent experiments. ^∗^*P* < 0.05 and ^∗∗^*P* < 0.01 *vs*. vehicle control group (one-way ANOVA with Tukey’s *post hoc* test).

To investigate whether trimebutine can inhibit cell migration, we performed a wound healing assay using U-87 MG human glioblastoma cells. As was shown in **Figure 2A**, no significant difference in the gap distance was found between each trimebutine-treated group and the vehicle control group 0 h after scratching. After 24, 48, and 72 h of incubation, 50, 100, and 200 μM of trimebutine treatment significantly impeded gap closure, with the most effective inhibition observed at 200 μM for all time points (**Figure 2B**). These results indicated that trimebutine can suppress glioma/glioblstoma cell migration.

### Trimebutine Inhibits Colony Formation of Glioma/Glioblastoma Cells

**FIGURE 3:**
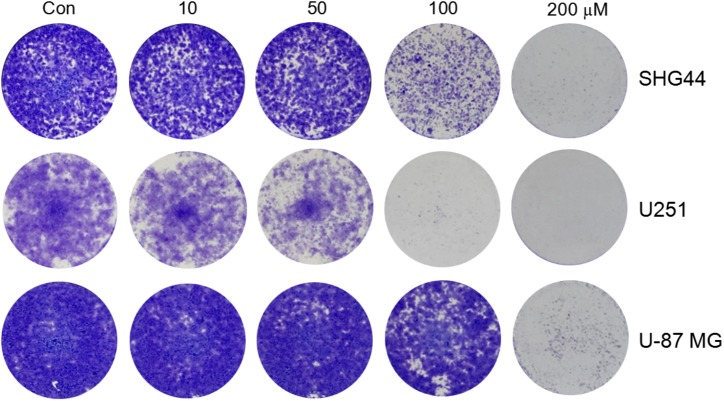
Trimebutine inhibits colony formation of glioma/glioblastoma cells. Representative images from 3 independent experiments for colony formation are shown. Trimebutine markedly inhibit colony formation in SHG44 and U251 human glioma and U-87 MG human glioblastoma cells.

To investigate whether trimebutine can inhibit cell colony formation, we performed a colony-formation assay using SHG44 and U251 human glioma and U-87 MG human glioblastoma cells. The results demonstrated that trimebutine can apparently suppress the colony formation in all three glioma/glioblastoma cell lines in a dose-dependent manner when compared with the control groups (**Figure 3**).

### Trimebutine Promotes Cell Apoptosis

**FIGURE 4:**
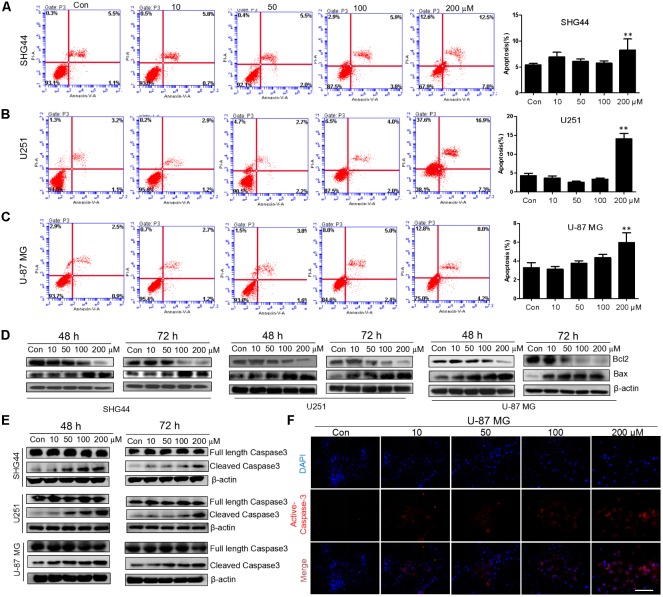
Trimebutine promotes cell apotosis in glioma cells. **(A–C)** Three cell lines were treated with 0, 10, 50, 100, and 200 μM of trimebutine for 48 h, and then stained with Annexin V-EGFP/PI. The percentage of apoptotic cells was determined using flow cytometry. The data are presented as the means ± SEM for 3 independent experiments. ^∗^*P* < 0.05 and ^∗∗^*P* < 0.01 vs. vehicle control group. **(D)** Western blot analysis showed the protein expression of Bcl-2 and Bax in three glioma/glioblastoma cell lines treated with 0, 10, 50, 100, and 200 μM of trimebutine for 48 and 72 h. DMSO was used as the vehicle control. **(E)** Western blot analysis showed the protein levels of both full length and cleaved Caspase-3 in three glioma/glioblastoma cell lines treated with 0, 10, 50, 100, and 200 μM of trimebutine for 48 and 72 h. DMSO was used as the vehicle control. **(F)** Photographs showing cells stained with the active Caspase-3 antibody (red) under a fluorescence microscope. Nuclei were counterstained with DAPI (blue). Scale bar, 200 μm.

To investigate whether trimebutine can lead to glioma cell apoptosis, we performed Annexin V-EGFP/PI Apoptosis assay. After 48 h treatment with either DMSO or trimebutine, apoptotic cells were examined through flow cytometry. The results demonstrated that trimebutine induced a significant apoptosis in SHG44 (**Figure 4A**), U251 (**Figure 4B**) human glioma and U-87 MG (**Figure 4C**) human glioblastoma cells when compared with the vehicle control. As was shown in **Figure 4D**, trimebutine dose-dependently reduced the protein level of Bcl-2, while increasing that of Bax in all three cell lines at both 48 and 72 h time points. In addition, trimebutine dose-dependently promoted the cleavage of Caspase-3 in all three glioma/glioblastoma cell lines at both 48 and 72 h time points (**Figure 4E**). Immunofluorescence staining also demonstrated that trimebutine can increase the level of activated Caspase-3 in a dose-dependent manner in U-87 MG glioblastoma cells (**Figure 4F**). These findings, combined with our previous data, indicated that trimebutine can induce apoptosis in human glioma/glioblastoma cells.

### Trimebutine Inhibits the ERK and AKT Signaling Pathways

**FIGURE 5:**
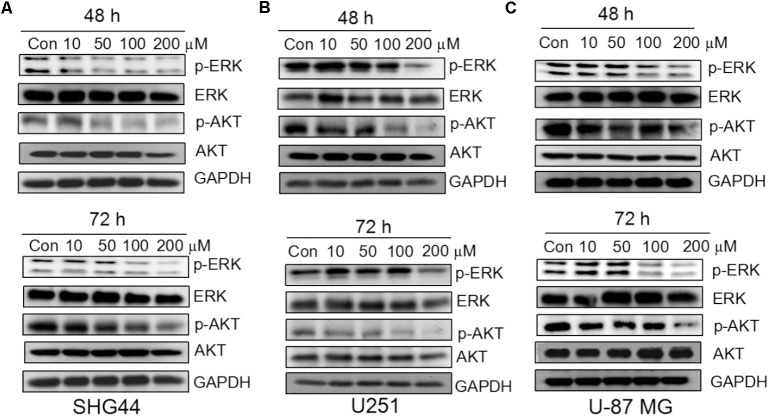
Trimebutine inhibits activation of ERK and AKT signaling pathways in glioma cells. **(A)** p-AKT and p-ERK protein levels were detected by western blot in SHG44 cells after treatment with various concentrations of trimebutine for 48 and 72 h. **(B)** p-AKT and p-ERK protein levels were detected by western blot in U251 cells after treatment with various concentrations of trimebutine for 48 and 72 h. **(C)** p-AKT and p-ERK protein levels were detected by western blot in U87 cells after treatment with various concentrations of trimebutine for 48 and 72 h.

To investigate whether trimebutine can prevent the growth of glioma/glioblastoma cells through the inhibition of p-ERK and p-AKT, western blot was performed to analyze the protein levels of p-ERK and p-AKT in SHG44, U251 andU-87 MG cells in response to trimebutine treatment (50, 100, and 200 μM) for 48 and 72 h. The results demonstrated that p-AKT levels were profoundly reduced after treatment with trimebutine at 50, 100, and 200 μM for 48 h in all three cell lines, whereas profoundly reduced p-AKT levels were detected in cells treated with trimebutine for 72 h at doses of 100 and 200 μM (**Figures 5A–C**). Similarly, p-ERK levels in each trimebutine-treated group were apparently reduced in a dose-dependent manner at both 48 and 72 h, with profound inhibition effect detected at 100 and 200 μM (**Figures 5A–C**).

### Trimebutine Reduces Tumorigenesis *in Vivo*

**FIGURE 6:**
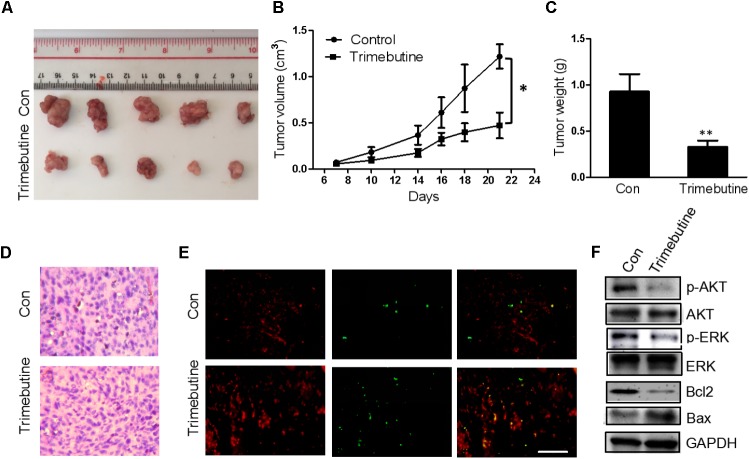
Trimebutine inhibits tumor growth in a U-87 MG glioblastoma xenograft model. **(A)** Representative photographs of the gross U-87MG xenograft glioblastoms from nude mice treated with trimebutine or vehicle control. **(B)** The tumor volume was evaluated before and after vehicle control/trimebutine treatment. Tumor weight was measured after dissection (^∗^*P* < 0.05 vs. vehicle control group). **(C)** The average final tumor weight in trimebutine-treated mice was significantly lower than that in DMSO-treated nude mice (^∗∗^*P* < 0.01 vs. vehicle control group). **(D)** Representative images for H&E staining from either group was shown. **(E)** Representative images for TUNEL-positive and PI-positive cells in the tumor tissue. TUNEL (green) and PI (red). Scale bar, 200 μm. **(F)** At the end of the experiment, tumor tissues were excised from mice, and the protein lysates extracted from the tumor tissues were assessed by western blot for p-AKT, AKT, p-ERK, ERK, Bcl-2, Bax, and GAPDH, respectively.

An *in vivo* xenograft nude mouse model was used to further confirm the findings that trimebutine can significantly reduce the glioma cell migration and colony formation. Nude mice subject to subcutaneous U-87 MG cell xenograft formation were treated with either DMSO or trimebutine for 7 days, and the tumor size was measured at 7, 10, 14, 18, 21 days. Dissected U-87 MG cell xenografts were shown in **Figure 6A**. The results showed that trimebutine treatment significantly inhibited glioblastoma xenograft formation of U-87 MG cells at each time point (**Figure 6B**). The average final tumor weight in trimebutine-treated mice was significantly lower than that in DMSO-treated mice (**Figure 6C**). H&E staining demonstrated a relatively loosened structure in the trimebutine-treated xenograft glioblastoma tissue (**Figure 6D**). Furthermore, TUNEL assay was used to evaluate the apoptotic cells in the tumor tissue. Significantly elevated apoptotic cells were observed in the trimebutine-treated glioblastoma tissue (**Figure 6E**). Western blot analysis demonstrated that both p-AKT and p-ERK levels were reduced in trimebutine-treated tumor tissues compared with the vehicle control (**Figure 6F**). In addition, trimebutine reduced the protein level of Bcl-2 while increasing that of Bax (**Figure 6F**). These data suggest that trimebutine may induce apoptosis via interfering with AKT and ERK signaling pathways.

## Discussion

Gliomas are the most common and malignant central nervous system tumors in human. Glioblastoma (GB), a high grade glioma, represents 46.6% of malignant central nervous system tumors in adults (Ostrom et al., 2016). Despite advanced surgery and radio-/chemotherapy have been developed for treatment of gliomas, the median overall survival (OS) is approximately 14 months (Stupp et al., 2009), and a 5-year survival rate is only 5.5% (Ostrom et al., 2016). The therapy of glioblastoma remains a challenge. Trimebutine maleate has proved efficient in treating irritable bowel syndrome (Delvaux and Wingate, 1997; Poynard et al., 2001), whereas there are no reported data about the effects of trimebutine on the function of glioma cells. Our present study demonstrated that trimebutine significantly reduced glioma/glioblastoma cell viability. We also showed that exposure of human glioma/glioblastoma cells to trimebutine obviously inhibited cell migration and colony formation. Our findings indicated that trimebutine may exert its anticancer functions by inhibiting the viability and migration of glioma/glioblastoma cells. By contrast, one previous study authenticated that trimebutine can significantly inhibit cell proliferation in human LOVO colon cancer cells at 100 μM (Jemel-Oualha et al., 2016). Notably, we observed differential response of three glioma/glioblstoma cells to trimebutine treatment. It has been reported that U-87 MG cell line contains a high percentage of glioblastoma stem cells, which may contribute to its resistance to trimebutine (Huang et al., 2010; Zhao and Schachner, 2013). Thus, a combined use of trimebutine and other therapeutic means targeting tumor stem cells may prove more effective in treating high-grade glioma.

Cell apoptosis is an improtant process involved in suppressing tumorigenesis (Hale et al., 1996). The apoptotic cell signaling exists in all human cells as an intricately regulated endogenous mechanism by which cells essentially undertake self-destruction (Degterev et al., 2003). This ability to undergo preprogrammed cell death has a role on several physiological processes. Anti-apoptotic proteins function as important physiological safeguards to prevent cellular destruction. These proteins also play a pathologic role in promoting the development of many malignant tumors, especially that of gliomas (Ziegler et al., 2008). Upregulation of the anti-apoptotic proteins Bcl-2 and Bcl-XL have been reported to contribute to the recurrence of glioblastomas, accompanied with the downregulation of pro-apoptotic Bax (Steinbach and Weller, 2004). In the preclinical studies using an antisense approach, downregulation of BCL-2 or BCL-XL resulted in glioma cell death and sensitization to the effects of chemotherapy and radiotherapy (Guensberg et al., 2002; Jiang et al., 2003; Zhu et al., 2003). In the present study, trimebutine dose-dependently induced cellular apoptosis, as was confirmed by flow cytometry with Annexin V-EGFP/PI staining. The apoptosis-promotion effect of trimebutine was further confirmed by marked reduction of Bcl-2 and significant increase of Bax. In contrast to the other two cell lines with lower malignancy, U-87 MG cells express less Bax. Trimebutine can increase Bax at a low level in U-87 MG cells, suggesting that it may counteract the proliferation of high-grade glioma cells by inducing the expression of Bax. We also found the increase of the number of active Caspase-3-positive cells in response to trimebutine. In human LOVO colon cancer cells, trimebutine can induce the presence of oligonucleosomal DNA fragmentation resulting in cell apoptosis (Jemel-Oualha et al., 2016). These combined data indicated that trimebutine can potently induce cell apoptosis in human glioma/glioblastoma cells in a dose- and time-dependent manner.

The Ras/MAPK/ERK and PI3K/AKT signaling pathways play crucial roles in multiple cancer cells and are highly related to tumor development (Sebolt-Leopold and Herrera, 2004; Vitucci et al., 2013; Tsai et al., 2015; Xiang et al., 2017; De Araújo et al., 2018). ERK is a member of MAPK family that is involved in many cellular functions, including cell growth, differentiation, survival and apoptosis (Sebolt-Leopold and Herrera, 2004). AKT is a key component in the PI3K pathway involved in cell survival, growth and cell apoptosis (Engelman, 2009; Pan et al., 2015; Yang et al., 2017). Our results showed that the levels of both p-AKT and p- ERK1/2 were decreased in response to trimebutine. Indeed, trimebutine has been shown to inhibit cell proliferation by suppressing the activation of the ERK signaling pathway in colon cancer cells (Jemel-Oualha et al., 2016). These data imply that trimebutine may inhibit the PI3K/AKT and MAPK/ERK signaling pathways to attenuate proliferation and promote cell apoptosis in glioma cells, thus leading to the inhibition of cell migration and colony formation.

To extend the observations *in vitro*, we performed the *in vivo* experiment to study the effects of trimebutine on the growth of human glioblastoma U-87 MG xenografts in a nude mouse model. Our results demonstrated that the application of trimebutine can significantly suppress the growth of the established glioblastoma xenografts *in vivo.* More TUNEL-positive apoptotic cells are observed in trimebutine-treated tumor tissues. Moreover, the molecules such as p-ERK, p-AKT, and Bcl-2 in tumor tissues were all down-regulated, whereas Bax was up-regulated in response to trimebutine treatment, which was consistent with *in vitro* results we observed. In coincidence with the *in vitro* results, trimebutine exhibited a profoundly effective inhibition of the glioma growth *in vivo* in the present study. Different from the intracerebral orthotropic implantation, no blood brain barrier (BBB) forms in the subcutaneuous glioma xenograft, which may increase drug utilization and therapeutic efficacy of trimebutine. It has been reported that intravenous administration of trimebutine can effectively attenuate the lesions of spinal cord in a mouse spinal cord injury (SCI) model (Xu et al., 2017), indicating that trimebutine can penetrate BBB and access targeted glioma cells. Further studies using the intracerebral orthotropic glioma xenograft model are warranted.

In summary, trimebutine can inhibit glioma/glioblastoma cell migration and proliferation, and promote cell apoptosis through regulating the MAPK/ERK and PI3K/AKT signaling pathways, suggesting that trimebutine may represent a novel therapy for malignant glioma.

## Author Contributions

Y-pF, H-cP and W-kX performed the research. H-cP and W-jZ designed the research study. Y-pF, H-cP, and PL analyzed the data. H-cP and W-jZ wrote and edited the paper.

## Conflict of Interest Statement

The authors declare that the research was conducted in the absence of any commercial or financial relationships that could be construed as a potential conflict of interest.
